# Income inequality and fear of crime across the European region

**DOI:** 10.1177/1477370816648993

**Published:** 2016-05-22

**Authors:** Christin-Melanie Vauclair, Boyka Bratanova

**Affiliations:** Instituto Universitário de Lisboa (ISCTE-IUL), Portugal; University of St Andrews, UK

**Keywords:** Cross-national comparisons, European Social Survey, fear of crime, income inequality, multilevel analyses, subjective well-being

## Abstract

This paper aims to take a holistic approach to studying fear of crime by testing predictors at multiple levels of analyses. Data from the European Social Survey (*N* = 56,752 from 29 countries) were used to test and extend the Income Inequality and Sense of Vulnerability Hypotheses. The findings confirm that (1) individuals in societies with greater income inequalities are more fearful of crime, and (2) older or disabled people as well as women report greater fear of crime. Contrary to the hypotheses, ethnic *majority* and not ethnic minority members report greater fear of crime, if they reside in high income inequality countries. It is further demonstrated that fear of crime explains the inverse association between income inequality and subjective well-being in this particular subsample.

Feeling safe can be seen as a basic human need (Maslow, 1970) that needs to be fulfilled to allow individuals to realize their full potential (Marmot, 2004). Not surprisingly, fear of crime research has become a central area of criminological investigation, as well as a key focus of crime policy throughout the world (Brunton-Smith and Sturgis, 2011). Despite its international relevance and the descriptive empirical evidence that average levels of fear of crime vary across countries (for example, Blinkert, 2010; Ceobanu, 2010; Hummelsheim et al., 2011; Semyonov et al., 2012), there is surprisingly little empirical research that aims to explain why people feel more fearful in some countries than in others.

To date, research has mainly focused on examining factors that may be associated with fear of crime in a piecemeal fashion. These studies can be distinguished in regard to the different levels of analyses that they address. Within the scope of individual-level studies, the focus has been on examining individual-level predictors and consequences of people’s fear of crime. Much research has been devoted to the vulnerability hypothesis, which stipulates that socio-demographic factors such as age, gender, ethnicity, and physical disability are predictors of fear of crime since people who belong to these social groups feel physically or socially vulnerable (Rader et al., 2012) and, therefore, at a higher risk of victimization (see, for example, Pain, 2000). Others have emphasized the role of (direct and indirect) victimization experience as an important factor in developing fear of crime (see, for example, Lane et al., 2014).

Fear of crime has wide-reaching consequences in regard to physical, psychological, behavioural, and social effects (see Lane et al., 2014). It is detrimental to people’s physical health and mental well-being and influences people’s behaviour, for example by engaging in crime prevention strategies such as locking doors and not walking alone at night. Although this may temporarily enhance a sense of security, it can also lead to social withdrawal and disengagement from activities in the community that are importantly related to subjective well-being. Given that fear of crime is primarily a psychological phenomenon, its consequences should be most prominent at the psychological level. A great deal of individual-level research has indeed corroborated that fear of crime is detrimental to people’s subjective well-being (for example, Cohen, 2008; Hanslmaier, 2013; Lane et al., 2014; Michalos and Zumbo, 2000; Moore, 2006).

In contrast to this individual-level research, the more sociological tradition has focused on the wider social context in which individuals reside in order to explain their fear of crime (Brunton-Smith and Sturgis, 2011). Most of this research has used data from the United States (see Lane et al., 2014) and examined the effect of context on fear of crime at the meso-level in the form of neighbourhood or local community characteristics. Although these studies have advanced our understanding of how context factors can explain variations in fear of crime, many of them are conditioned by the US context and do not provide any insights into macro-level variations in fear of crime. The few studies that have examined fear of crime across macro-level units, such as nations, have focused on examining either antecedents or consequences of fear of crime and mostly in a rather atheoretical fashion (for example, Blinkert, 2010; Ceobanu, 2010; Hummelsheim et al., 2011; Semyonov et al., 2012; Vieno et al., 2013). Although these studies usually analysed predictors at both the individual and country level, they did not examine more theory-guided multilevel models with mediating variables or how country-level factors may moderate individual-level associations (so-called cross-level interactions).

In this paper, we aimed to test an influential theory in the social science literature that stipulates that income inequality yields a number of psychological issues, including a greater fear of crime and worse subjective well-being. We tested this hypothesis by adopting a holistic perspective, that is, by examining both antecedents and consequences of fear of crime taking into account who is most vulnerable to this issue. In order to achieve this aim, we used representative data from the European Social Survey across 29 countries. We first tested the robustness of income inequality as a predictor of fear of crime and examined it then as a cross-level moderator for individual-level associations with fear of crime. The aim was to examine the vulnerability hypothesis in context, that is, whether a context of greater income inequality also renders vulnerable social groups more susceptible to fear of crime. We then analysed a mediation model in which fear of crime is specified as the mediator for the income inequality–subjective well-being link. Throughout these analyses, we used multilevel modelling techniques in order to take into account the clustered data structure and to reduce the probability of Type-I errors. We used the most recent developments in multilevel structural equation modelling in order to conduct the mediation analyses (Preacher et al., 2011). By adopting this type of methodology, we are able to test a comprehensive theoretical model about the causes and effects of fear of crime. To the best of our knowledge, a similar theoretical model has already been suggested elsewhere (Lorenc et al., 2012) but never tested with cross-national data yet.

## Income inequality as an antecedent and moderator

Wilkinson and Pickett (2009) argued that income inequality is the most parsimonious explanation for a host of social and health problems including the fear of crime. They show that there is a strong and consistent association between inequality and violence (for example, in terms of homicides and assaults) across different time periods and settings and argue that inequality is a form of ‘structural’ violence triggered through the great gap in income. From a socio-psychological point of view, inequality translates into highly hierarchical societies in which the attainment of status becomes an important goal in individuals’ lives. While status competition increases, the income gap makes it increasingly difficult for the poorer to obtain markers of status and social success (for example, in terms of a good education, nice houses, good jobs). Ultimately, the response to this form of frustration and humiliation is violence, which in turn fuels the fear of crime in the general population.

The authors also point out that people’s fears of crime do not always match up with rates and trends in crime and violence. It is conceivable that income inequalities rather foster a climate of fear because people believe that there are only a few legitimate chances for the deprived to obtain resources and social status, leaving crime and violence as the most obvious route. Consequently, the widely shared perception is that those who are most deprived are potentially dangerous even if this does not always match the actual evidence. This perception should be heightened for those who belong to a social group that is commonly seen as an ‘easy’ crime target (we expand on this below).

A few cross-national studies have indeed found that economic inequality is positively linked with fear of crime (Hummelsheim et al., 2011; Vieno et al., 2013). The argument that has been put forward is that income inequality signals less social protection by increasing the general concern about the erosion of the social and moral order, resulting in greater fear of crime. Hence, fear of crime can also be seen as an ‘umbrella sentiment’ that expresses socio-economic insecurities rather than actual crime-related insecurities (Vieno et al., 2013).

## Alternative macro-level explanations

Besides income inequality, there are other macro-level factors that may fuel people’s fear of crime. Theorists within the sociological literature have long argued that a better understanding of variations in individual-level fear of crime requires incorporating the context in which individuals reside. Traditionally, these context variables are located at the meso-level by describing the neighbourhood and community characteristics in which individuals live (for a recent review, see Lorenc et al., 2014). A great deal of research has been devoted to studying meso-level factors, whereas very little research has examined the macro-level context and its relation to fear of crime (Hummelsheim et al., 2011). This is even more surprising given that empirical evidence has shown that fear of crime varies across countries. For instance, within Europe it seems that Southern and East European countries as well as the United Kingdom show greater fear of crime (for example, Blinkert, 2010; Ceobanu, 2010; Hummelsheim et al., 2011; Semyonov et al., 2012; Vieno et al., 2013; Visser et al., 2013). This opens up the question of which macro-level factors explain why individuals are more fearful of crime in some countries than in others.

One of the most obvious explanations is that greater crime rates should produce greater fear of crime in the population. If individuals live in an environment that provides them with information about crime rates, they should process this information into their risk perceptions, leading to greater fear of crime (Lorenc et al., 2012). Yet one recent macro-level study using cross-national data from the European region found that registered crime rates actually had little explanatory power in explaining fear of crime (Hummelsheim et al., 2011).

Other common explanations centre on the argument that fear of crime is fuelled by (1) *economic characteristics* such as economic disadvantage (for example, Franklin et al., 2008; Roman and Chaflin, 2008); (2) *structural characteristics* such as immigration concentration, which can trigger feelings of intimidation and threat posed by the migrants (for example, Sampson and Raudenbush, 2004; Wang, 2012), and (3) *social characteristics* such as social capital, which in its absence fuels fear of crime because there is a lack of social monitoring that could deter crime (see Sampson, Raudenbush, and Earls, 1997) and rouse a sense of security in residents (for example, Ferguson and Mindel, 2007; Lindström et al., 2003; Roman and Chalfin, 2008).

A few macro-level studies have examined these explanations using archival data with representative samples from countries in the European region. What becomes clear is that there is some ambiguity in regard to the operationalization of the economic disadvantage factor. Some studies find a significant association between (un)employment and fear of crime (for example, Hummelsheim et al., 2011), whereas others do not find this link (Visser et al., 2013). Wealth did not emerge as a significant predictor of fear of crime in Ceobanu’s (2010) study; however, social protection expenditures seem to be consistently predictive of fear of crime (Vieno et al., 2013; Visser et al., 2013; Hummelsheim et al., 2011).

Regarding the operationalization of structural characteristics such as immigration concentration, this has usually been assessed by the absolute or relative number of foreigners in a country. Some of the macro-level studies suggest that the size of the migrant population in a country is related to heightened fear of crime (for example, Ceobanu, 2010; Semyonov et al., 2012), yet again other studies did not find this link at all (Visser et al., 2013).

Finally, social capital can be examined as a social characteristic of countries predicting fear of crime. Although there is a lack of cross-national studies in this regard, meso-level studies have shown that a lack of social capital arouses individuals’ fear of crime.

Overall, it is difficult to draw clear conclusions based on the few available studies because they all used different cross-national datasets from different sources (for example, Eurobarometer, European Social Survey) and for different periods of time (from 2002 to 2008). Moreover, given the different foci in each study, a number of covariates were either included or not when assessing the impact of the macro-level context on fear of crime, which can also alter the results.^1^ In this study, we aimed to integrate previous research by testing whether income inequality holds as a predictor of fear of crime when controlling for relevant *socio-economic, structural* and *social macro-level characteristics*. Hence, we hypothesized that:

**H1:** Individuals living in countries with higher income inequality report more fear of crime than individuals residing in low inequality countries even after controlling for alternative macro-level explanations.

## Personal vulnerabilities as micro-level predictors

Numerous studies have sought to identify who is most fearful of crime and why this is the case, and this has culminated in the Sense of Vulnerability Hypothesis (for example, Cossman and Rader, 2011). The idea is that some social groups in society are more fearful because they believe that they are at higher risk of victimization since they would be unable to defend themselves should a criminal attack occur.

Gender and age have been the most studied markers of physical vulnerability to fear (Hale, 1996; Killias, 1990). Although victimization rates are higher among males than females (Smith and Torstensson, 1997), the latter appear to be more fearful of crime because they feel less capable of physically defending themselves, they have lower perceived self-efficacy, and they perceive themselves and their social group as more likely to become victims. This so-called victimization–fear paradox has also been found to exist in relation to age, where older people reported consistently higher levels of fear and worry about crime and safety issues despite relatively low victimization rates in this age group (Hale, 1996). Their fear is again grounded in the perception of control over the event and the subsequent consequences for themselves. Individuals who are physically impaired are another social group that fits into this category (for a review, see Pain, 2000).

Although gender and age have been widely studied in the fear of crime literature (see Hale, 1996), the association between the level of reported physical limitations and fear of crime has been largely neglected (Taylor et al., 2009). Yet there is evidence that a physical disability increases the likelihood of being a victim of crime (for example, Rosen, 2006). One of the few studies examining this issue found that having a physical disability was a significant predictor of fear of crime among young adults in the US (Stiles et al., 2003). Hence, the authors concluded that the *physical vulnerability hypothesis* is also applicable to those who report physical disabilities. They argue that individuals with physical limitations may feel a sense of helplessness or vulnerability, and consequently fear to find themselves in actually or potentially harmful situations in which they have limited behavioural options in regard to fleeing or protecting themselves (see also Taylor et al., 2009, for another study in the US). Pain (2000) also reports a study from the United Kingdom in which physical disability emerged as a marker for feelings of vulnerability and avoidance strategies in order to deal with fear of crime. Despite this scattered evidence, it is still not clear whether the association between physical disability and fear of crime occurs across different national contexts.

Besides physical vulnerability characteristics (such as age, gender, and disability), there are also social vulnerability characteristics (Rader et al., 2012) that have been argued to be important in determining one’s fear of crime. One of the most prominent social vulnerability descriptors is race or ethnicity. The few studies that examined the race–fear relationship are predominantly from the US. In this context, researchers have found that ethnic minorities were more fearful of crime than Caucasians (see Lane et al., 2014), which may be explained by the fact that ethnic minority groups show significantly higher rates of victimization because of their racial or ethnic background (Pain, 2000). Consequently, they feel vulnerable to and fearful of racist attacks and harassment over which they have little control and which can lead to severe consequences. Studies from the European countries show less consistent associations between ethnic minority membership and fear of crime (Visser et al., 2013).

In this study, we will re-examine the vulnerability hypothesis in relation to age, gender, disability, and ethnic minority membership. We hypothesized that:

**H2:** Females, ethnic minorities, older individuals, and individuals with a disability are more fearful of crime.

We also explore the joint effects of the vulnerability and income inequality hypotheses, that is, we examine whether individuals belonging to vulnerable social groups report greater fear of crime in countries characterized by greater income inequality. Given that income inequality generally heightens fear of crime, it is conceivable that this worry is exacerbated in more vulnerable groups living in high inequality societies:

**H3:** Income inequality moderates the association between vulnerable social group membership and fear of crime. Individuals belonging to vulnerable social groups feel more fearful in high than in low inequality countries.

## Subjective ill-being as a consequence

Subjective well-being is comprised of cognitive and affective components. The cognitive component is usually measured as a global evaluation of life satisfaction (Diener et al., 1995). The affective component, or emotional well-being, is usually assessed by evaluations of happiness (see Diener et al., 1995). Although happiness and life satisfaction tap into separate components theoretically, they seem to have similar correlates with fear of crime and in fact have been found to be highly correlated empirically in data from the European Social Survey (for example, Swift et al., 2014). Previous studies have consistently shown that fear of crime is negatively associated with the components of subjective well-being (Hanslmaier, 2013; Michalos and Zumbo, 2000; Moore, 2006; Morrall et al., 2010).

Not only individual-level variables such as fear of crime can predict an individual’s subjective well-being, but so can contextual factors. There is evidence that income inequality is negatively associated with subjective well-being (for example, Diener et al., 1995; Hagerty, 2000). Recently, some researchers aimed to identify a psychological mechanism that accounts for this link and they found that generalized trust explained the income inequality–subjective well-being link in the US. In this paper, we propose fear of crime as an alternative psycho-social pathway that may explain the inequality–subjective well-being nexus. Whereas generalized trust is a relatively diffuse variable that is rather difficult to tackle through social interventions and policy directives, fear of crime can be regarded as a more concrete manifestation of distrust in generalized others (Wilkinson and Pickett, 2009). Hence, we hypothesized that:

**H4:** Individuals show lower levels of subjective well-being in unequal societies compared with more equal societies and this association is explained by their fear of crime.

## Methods

### Data source

We used data from the European Social Survey (ESS) from Round 4, 4th edition (European Social Survey Round 4 Data, 2008). The data were collected through computer-based personal interviews in 29 countries (*N* = 56,752) from the European region, plus Israel, in the years 2008 to 2010. They are based on random probability samples and are nearly representative of the eligible residential populations in each country aged 15 years and over (*M*_age_ = 47.54, *SD* = 18.50; 54.5% female).

### Individual-level variables

#### Fear of crime

The operationalization of the concept fear of crime has been open to much debate. This is partly due to the fact that it is multidimensional in nature (Ferraro and LaGrange, 1987) and that there has been a confusion of cognitive (for example, risk assessments), affective (fears and worries), and behavioural dimensions (for example, security precautions) in fear of crime assessments (see Fattah and Sacco, 1989). Although a full consensus has not been reached to date, it is widely acknowledged that fear, and therefore also ‘fear of crime’, is primarily an emotion (Lane et al., 2014), that is, an emotional response to a perceived threat. Hence, a standard indicator of fear of crime has been the question of how safe the respondent would feel if walking alone in the area after dark (Hale, 1996). According to Fattah and Sacco (1989), this item is a global measure of the affective component of fear of crime. More concrete measures include questions involving worry or fear of becoming a victim of specific criminal offences.

Hence, we decided to use the following ESS items as measures of fear of crime: ‘How safe do you – or would you – feel walking alone in this area after dark?’ (1 = ‘very safe’, 4 = ‘very unsafe’), ‘How often, if at all, do you worry about your home being burgled?’ (1 = ‘all or most of the time’, 4 = ‘never’), and ‘How often, if at all, do you worry about becoming a victim of violent crime?’ (1 = ‘all or most of the time’, 4 = ‘never’). The latter two items were recoded so that higher scores indicate greater fear of crime. We computed an overall index of fear of crime by averaging the scores on these three variables.

Ferraro and LaGrange (1987) suggested that ‘formless’ fears, as in the safety question, may not just tap into the affective component of fear of crime, but can also be seen as judgements about the likelihood of criminal victimization for the individual. And, therefore, they tap into a more cognitive component of fear of crime and may produce different results compared with concrete questions about fears. In order to gauge whether these items do indeed differ from each other empirically, we conducted a principal component analysis (PCA) using the eigenvalues >1 criterion. The results showed that all three items loaded on the same factor (explaining 63 percent of the variance) with loadings above .72. Cronbach’s alpha was satisfactory with a value of .71 and would not have increased much if the item ‘feeling of safety’ was deleted (α = .72). Considering the low number of items, Cronbach’s alpha was also satisfactory across countries, ranging from .51 (Belgium) to .82 (Greece). A PCA conducted separately in each country also corroborated that there was only one factor onto which the three items loaded. Hence, we are confident that in this case it is justified to use an index composed of these three items, which represents the affective component of fear of crime across countries.

#### Subjective well-being

As a measure of subjective well-being, we used two 11-point Likert scaled items that asked respondents ‘All things considered, how satisfied are you with your life as a whole nowadays?’ (0 = ‘extremely dissatisfied’ to 10 = ‘extremely satisfied’) and ‘Taking all things together, how happy would you say you are’ (0 = ‘extremely unhappy’ to 10 = ‘extremely happy’). The inter-item correlation was satisfactory for all countries, ranging from .49 (Turkey) to .76 (Sweden). We computed an overall index of subjective well-being by averaging the scores on these two variables.

#### Socio-demographics

Individual-level variables indicating perceived vulnerability to becoming a victim of crime were *age*, *gender* (dummy coded: 0 = male, 1 = female), *ethnic minority membership* (dummy coded: 0 = no, 1 = yes), and *disability* (‘Are you hampered in your daily activities in any way by any longstanding illness, or disability, infirmity or mental health problem?’; recoded into: 1 = no, 2 = yes to some extent, 3 = yes a lot).

Guided by previous research (for example, Hummelsheim et al., 2011; Pain, 2000), we included the following measures from the ESS as socio-demographic covariates in the model since they have been found to be predictors of fear of crime: years of full-time *education* completed, in *paid work* (that is, ‘employee, self-employed, working for own family business for the last 7 years?’; 0 = no, 1 = yes), *domicile* (1 = a big city, 2 = suburbs or outskirts of big city, 3 = town or small city, 4 = country village, 5 = farm or home in countryside; recoded so that higher numbers correspond to more urban domiciles), and *victimization* in the past 5 years (‘Have you or a member of your household been the victim of a burglary or assault in the last 5 years’?; dummy coded: 0 = no, 1 = yes).

### Country-level variables

#### Income inequality

As a measure of income inequality in countries, we used the Gini coefficient from Eurostat for the year 2008.^2^ The Gini coefficient for Turkey was available only for the year 2006. We complemented missing data in Eurostat with Gini coefficients from the World Income Inequality Database^3^ for Israel (from 2001), Russia (from 2006), and Ukraine (from 2006).

#### Socio-economic development

We used the Human Development Index (HDI) for the year 2007 (Human Development Report, 2009) as a proxy for the socio-economic development of a country. The HDI is a well-established indicator assessing life expectancy at birth, educational attainment, and Gross National Income per capita within a single statistic.

#### Total recorded crime

We used data from Eurostat on total crimes per thousand population reported by the police (that is, any offences against the penal or criminal code) in the year 2007.^4^ Data for Ireland were available only for the year 2006 and were not available at all for Israel, Russia, and the Ukraine.

#### Aggregated individual-level variables

In order to control for other relevant macro-level variables, we aggregated the following ESS individual-level variables at the country level: *generalized trust*^5^ (as a measure of social capital), *paid work* (percent as a measure of employment), *ethnic minority membership* ( percent as an assessment of immigration concentration), and *domicile* (as a measure of levels of urbanization^6^). Although we are dealing with representative samples, we are aware that a better strategy would have been to find external data on these variables. However, it is very difficult to find macro-level statistics for all ESS countries and, therefore, external data would have reduced our already limited country-level sample size even further.

### Statistical analysis

Given the clustered data structure (individuals nested within countries), we used multilevel regression analyses (with HLM 7.01, Raudenbush and Bryk, 2002). The aim was to examine whether there is a robust association between income inequality and fear of crime and whether the vulnerability hypothesis is context dependent, that is, whether social minorities are more fearful of crime in countries characterized by high income inequality. Our modelling strategy consisted of the following steps: Model 0 is the null model, without any predictors, which determines how much of the total variance in fear of crime is associated with country differences as opposed to individual differences (represented by the Intraclass Correlation Coefficient, ICC). In Model 1 we added income inequality as a country-level predictor of fear of crime. In subsequent analyses, we added one country-level covariate at a time in order to test the robustness of the inequality–fear of crime link while preserving a maximum degree of freedom in the analysis. Since sample size at the country level is relatively small (*N* = 29), we applied one-tailed significance level tests to the country-level predictors.

In Model 2, we examined whether the income inequality–fear of crime link remained significant when controlling for individual-level socio-demographics by *grand*-mean centring them (see Enders and Tofighi, 2007).

For Model 3, we used *group*-mean centring for all individual-level predictors because it removes all country-level variation from the predictor variable and yields slope coefficients that can be unambiguously interpreted as the pooled within-cluster regression of the respective predictor on fear of crime. This is the most appropriate centring strategy when associations between predictors and outcome variables at the lowest level are of substantive interest and is also preferable for examining cross-level interactions (Enders and Tofighi, 2007). We first added individual-level predictors with fixed slopes (Model 3a). We then examined whether the associations between vulnerable social group membership and fear of crime varied randomly across countries (Model 3b). Provided that these analyses yielded any significant random slopes, we examined in Model 3c whether income inequality explains country differences in these individual-level associations. In other words, we examined whether the vulnerability hypothesis is context dependent, meaning that we tested whether income inequality acts as a contextual moderator for the link between vulnerable social group membership (regarding age, gender, ethnic minority, disability) and fear of crime (referred to as cross-level interaction).

In the final set of analyses, we used multilevel structural equation modelling (MSEM) with the software Mplus 4.2 (Muthén and Muthén 2007) in order to test for the mediating effect of fear of crime on the income inequality–subjective well-being link. We created a 2-1-1 multilevel mediation model (see Preacher et al., 2011), meaning that the predictor (income inequality) is assessed at Level 2, and both the mediator (fear of crime) and the dependent variable (subjective well-being) are measured at Level 1. Hence, we expected that income inequality as a Level-2 antecedent influences the Level-1 mediator fear of crime, which then affects the Level-1 outcome subjective well-being.

Several procedures have been suggested for testing multilevel mediation within the standard multilevel modelling (MLM) framework. Yet, in the case of a 2-1-1 mediation, MLM does not fully separate a between-cluster and within-cluster effect, which means that it can introduce a bias in the estimation of the indirect effect and lead to very high Type-I error rates (Zhang et al. 2008). Although our focus is on the between-cluster relationships – because any mediation of the effect of a Level-2 variable must also occur at the between-cluster level regardless at which level the mediator and outcome variable are assessed – it is important to differentiate the relationships at the two levels rather than combining them into a single estimate within the indirect effect. One option that has recently been developed is a mediation analysis within the MSEM framework (Preacher et al., 2011). MSEM provides unbiased estimates of the between-group indirect effect by treating the cluster-level component of the Level-1 variable as latent.

Note that we used design weights (as provided by the ESS) in all analyses in order to adjust for a possible sampling bias.

## Results

Estimates of the multilevel regression models are summarized in Table 1.

**Table 1.** Multilevel regression analyses predicting individuals’ fear of crime.

	Models			
	0	1	2	3c
Intercept	**1.877**	**1.876**	**1.884**	**1.882**
*Individual-level predictors*				
Age			**0.001**	**0.001**
Gender (Female)			**0.234**	**0.233**
Disability			**0.096**	**0.094**
Ethnic minority membership			−0.013	0.010
Education			**−0.004**	**−0.004**
Paid work			0.006	0.004
Urban domicile			**0.059**	**0.058**
Crime victim (past 5 years)			**0.312**	**0.311**
*Country-level predictors*				
Income inequality		**0.012**	**0.010**	**0.013**
*Cross-level interactions*				
Income inequality * Age				−2.00E-05
Income inequality * Gender (Female)				−0.002
Income inequality * Disability				0.003
Income inequality * Ethnic minority membership				**−0.009**
*Variance components*				
Individual level (*r*)	0.398	0.398	0.358	0.355
Country level (*u*0)	**0.033**	**0.027**	**0.023**	**0.026**
Age (*u*1)				**0.000**
Gender (*u*2)				**0.004**
Disability (*u*3)				**0.001**
Ethnic minority membership (*u*4)				**0.006**
*Model fit statistics*				
Deviance (df)	97022(3)	97017(4)	87593(12)	87300(30)
*Explained variance*				
Individual level (percent)	–	–	10.10	10.89
Country level (percent)	–	16.59	30.06	20.01

*Note*: Analyses are based on 26 countries owing to the exclusion of Slovakia, Greece, and Bulgaria as outliers. Individual-level predictors are group-mean centred for Model 3c; all other predictors in the models are grand-mean centred. Bold estimates indicate significance at *p* < .05 (one-tailed).

### Income inequality and fear of crime

The ICC showed that a large proportion of the total variance in respondents’ fear of crime was due to individual differences (90.9 percent). Consequently, 9.1 percent of the total variance was associated with differences between countries and the chi-square statistic indicated that the differences in mean scores were significant (*χ*^²^(28) = 5708.13, *p* < .001), justifying follow-up analyses using country-level predictors.

**Figure 1.**
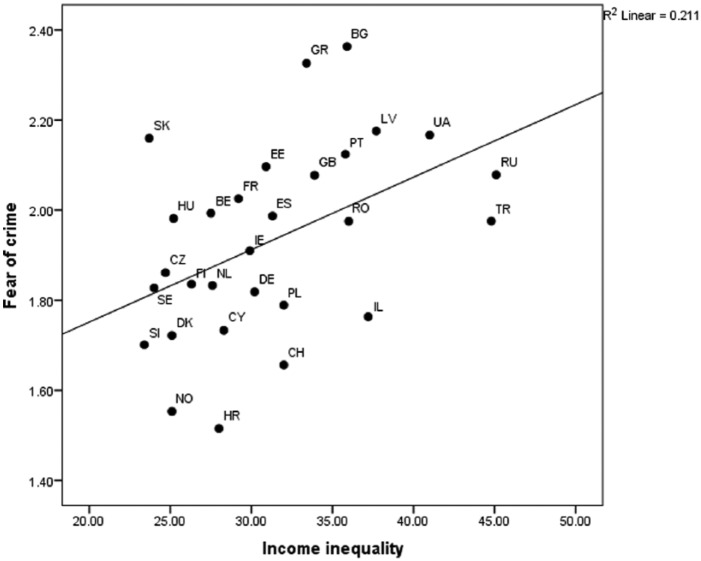
Scatter plot and best-fitting regression line showing average fear of crime scores in ESS countries as a function of income inequality. *Notes*: Belgium (BE), Bulgaria (BG), Switzerland (CH), Cyprus (CY), Czech Republic (CZ), Germany (DE), Denmark (DK), Estonia (EE), Spain (ES), Finland (FI), France (FR), United Kingdom (GB), Greece (GR), Croatia (HR), Hungary (HU), Ireland (IE), Israel (IL), Latvia (LV), Netherlands (NL), Norway (NO), Poland (PL), Portugal (PT), Romania (RO), Russian Federation (RU), Sweden (SE), Slovenia (SI), Slovakia (SK), Turkey (TR), Ukraine (UA).

Consistent with our hypothesis (H1), income inequality predicted fear of crime (Model 1). Figure 1 shows the association between income inequality and country-level scores of fear of crime, *r*(28) = .46, *p* < .05. The scatter plot also shows that three countries score relatively high on the fear of crime measure, yet they have only low or medium levels of income inequality. Hence, these countries (Slovakia, Greece, and Bulgaria) appeared to be outliers. This was also corroborated by a higher correlation between income inequality and fear of crime when these three countries were excluded from the analyses, *r*(25) = .55, *p* < .01. Hence, we decided to run the following multilevel analyses without these outliers.

Next, we proceeded to testing the robustness of the income inequality–fear of crime link by including relevant country-level covariates one by one in order to preserve a maximum degree of freedom at the country level. The effect of income inequality remained significant, *B* = 0.019, *SE* = 0.008, *p* = .01, when controlling for the total crime rates per country, which was not a significant predictor of fear of crime, *B* = 3.00E-06, *SE* = 1.60E-05, *p* = .49. In a similar vein, we found that the HDI was not a significant predictor of fear of crime, *B* = −0.074, *SE* = 0.086, *p* = .47, yet the effect of income inequality remained (marginally) significant, *B* = 0.012, *SE* = 0.001, *p* = .07. The social capital variable was also a non-significant predictor of fear of crime, *B* = 0.006, *SE* = 0.042, *p* = .45, and did not diminish the predictive power of income inequality, *B* = 0.013, *SE* = 0.007, *p* = .04. The model containing the covariate ‘proportion of people who are in paid work’ per country was not significantly related to fear of crime, *B* = −8.50E-05, *SE* = 0.004, *p* = .49, and had no impact on the inequality–fear of crime association, *B* = 0.012, *SE* = 0.006, *p* = .04. Moreover, income inequality remained a marginally significant predictor of the criterion variable, *B* = 0.011, *SE* = 0.008, *p* = .07, when controlling for the proportion of ethnic minorities in a country, which was not predictive of fear of crime, *B* = 0.002, *SE* = 0.008, *p* = .41. In the last country-level covariate model, we examined whether the level of urbanization in a country affects the relationship between income inequality and fear of crime. We found that income inequality remained a significant predictor, *B* = 0.017, *SE* = 0.006, *p* = .01, and urbanization was not predictive of fear of crime, *B* = 0.126, *SE* = 0.107, *p* = .13. In sum, these analyses point to the robustness of the income inequality–fear of crime link since it cannot be fully accounted for by a third variable.

Model 2 shows that the effect of income inequality remained significant after controlling for all individual-level socio-demographic variables. Moreover, we found that the vulnerability hypothesis was confirmed for some social groups, showing that women, older, and disabled respondents reported greater fear of crime (H2). Surprisingly, ethnic minority membership turned out to be a non-significant predictor of fear of crime. As expected, respondents who had been a victim of crime (or knew someone close who had been a victim in the past 5 years) were more fearful than those who did not have this experience. Consistent with previous research (for example, Hummelsheim et al., 2011; Pain, 2000), respondents who are less educated and living in urban areas were more fearful. However, the variable *paid work* was not a significant predictor of fear of crime in our analyses. The model explained 10.1 percent of the within-country variance.

In the next analysis step (Model 3a), we entered the *group*-mean centred fixed slopes of the individual-level covariates, which yielded virtually the same results as in Model 2. We then let the slopes of the predictors that indicate vulnerable social group membership vary randomly (Model 3b) and found that there was indeed considerable variation across countries (age: *χ*^²^(25) = 187.43, *p* < .001; gender: *χ*^²^(25) = 160.44, *p* < .001; ethnic minority membership: *χ*^²^(25) = 128.95, *p* < .001; disability: *χ*^²^(25) = 54.92, *p* = .001). We proceeded with testing whether income inequality can account for the significant slope variation of these individual-level predictors. Model 3c in Table 1 shows that there is only one significant cross-level interaction (H3), that is, the association between the variable ethnic minority membership and fear of crime is significantly moderated by income inequality. A chi-square test for nested models yielded a significant difference from Model 3b (*χ*^²^(4) = 13.00, *p* < .05), indicating that the random slope model with the cross-level interaction fitted the data better than the random slope model without the cross-level interaction.

**Figure 2.**
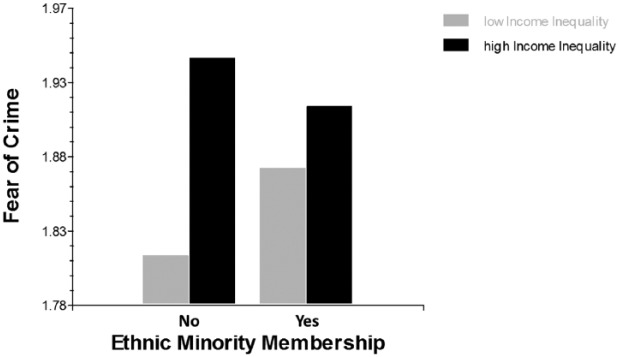
Cross-level interaction between income inequality and ethnicity in the prediction of fear of crime. *Note*: Low and high income inequality correspond to the averaged upper and lower quartiles.

Figure 2 depicts the results of the cross-level interaction. What can be seen is that ethnic majority members report the greatest fear of crime in high income inequality countries.

### Mediation model

For the mediation analyses, we used all 29 countries in order to enhance the sample size, which did not make any difference for the results. Pearson correlation coefficients showed that all variables in the mediation model correlate significantly with each other at the country level in the hypothesized direction. Income inequality correlates with subjective well-being at *r* = −.61, *p* < .001, and therefore it shares 37 percent of the variance with individuals’ subjective well-being. There is a relatively strong country-level correlation between fear of crime and subjective well-being, *r* = −.67, *p* < .001.

The total effect of the mediation analyses showed a significant negative association between income inequality and subjective well-being, *B* = −0.090, *SE* = 0.022, *Z* = −4.146, *p* < .001. As expected, on average respondents perceived their well-being to be worse in countries with more income inequality than in countries with less inequality. We next tested whether fear of crime mediates the association between income inequality and subjective well-being. We found that respondents reported more fear of crime in more unequal countries than in more equal ones, *B* = 0.012, *SE* = 0.007, *p* = .03. When taking into account fear of crime as a mediator in the third step, we found that greater fear of crime is associated with less subjective well-being at the country level, *B* = −1.974, *SE* = 0.621, *Z* = −3.179, *p* < .001, as well as the individual level, *B* = −0.386, *SE* = 0.029, *Z* = −13.166, *p* < .001. However, the negative relationship between income inequality and subjective well-being was not significantly reduced after including the mediator (H4), *B* = −0.066, *SE* = 0.020, *Z* = −3.323, *p* < .001, and the indirect effect was not significant, *B* = −0.024, *SE* = 0.016, 90% CI [−0.049, 0.002].^7^

**Figure 3.**
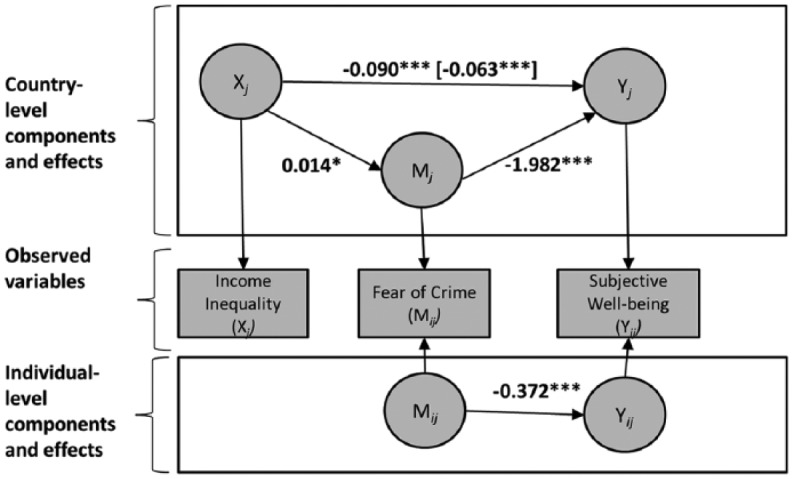
Multilevel structural equation model for a 2-1-1 mediation showing the association between income inequality and subjective well-being as mediated by fear of crime in a subsample of ethnic majority members. *Notes*: Regression coefficients are unstandardized; the coefficient in brackets is the direct effect. The figure is adapted from Preacher et al. (2011). *p < .05, **p < .01, ***p < .001 (one-tailed).

We followed up our previous finding on the cross-level interaction between ethnic minority membership and income inequality and explored whether the mediation model may hold only for respondents who are ethnic majority members. Figure 3 provides an overview of the results. The indirect effect is now significant, *B* = −0.027, *SE* = 0.016, 90% CI [−0.052, −0.001]. In contrast, running the same model for ethnic minority members shows that the association between income inequality and fear of crime becomes non-significant, *B* = −0.001, *SE* = 0.008, *Z* = −0.119, *p* = .905, as does the indirect effect, *B* = 0.002, *SE* = 0.015, 90% CI [−0.023, 0.026]. Hence, our mediation hypothesis holds only for ethnic majority members.

## Discussion

The main aim of this paper was to adopt a more holistic perspective on fear of crime by studying its antecedents at multiple levels of analysis as well as its psychological consequence. We examined the relationship between income inequality and fear of crime in representative samples from 28 countries in the European region, plus Israel. The evidence provides new insights into the phenomenon of fear of crime in regard to who is most affected by it and in which societal circumstances. First, it confirms the income inequality hypothesis (Wilkinson and Pickett, 2009), that is, that greater income inequality is associated with more fear of crime in the population. This association remains significant even if the effect of crime rates or the *socio-economic, structural* and *social* characteristics of a country, as well as relevant individual-level predictors (for example past victimization), are taken into account. Although the use of official crime rate statistics has some limitations, for example in regard to cross-national comparability, the finding suggests that fear of crime is triggered not just by actual levels of violence but by a system of income differences that fosters beliefs that violence is a likely and viable route to make up for status deprivation. This is also consistent with some findings that people’s fears of crime do not always match up with rates and trends in crime and violence (Ceobanu, 2010; Wilkinson and Pickett, 2009).

The findings are consistent with the vulnerability hypothesis (for example, Cossman and Rader, 2011) by showing that social groups that are usually perceived to be vulnerable to criminal attacks also report greater fear of crime. This was the case for older and disabled people as well as for women, yet not for ethnic minority members. We extended the vulnerability hypothesis by combining it with the inequality hypothesis and expected that vulnerable social groups may report greater fear in more unequal societies. Although the association between vulnerable social group membership and fear of crime differed significantly across countries, this variation could not be explained with income inequality. Future research may examine whether other macro-level variables account for these slope variations. Another explanation for this result may also be that cross-level interactions require greater cluster-level sample sizes in order to detect significant effects of smaller effect sizes. It might also be that income inequality does not affect *physically* vulnerable groups in their fear of crime more than others in society because everyone in an unequal society is affected by a social climate in which status competition and relative deprivations stir anxieties about crime and violence. Hence, being physically fit is not perceived as a protective factor any more.

However, we did find one significant cross-level interaction regarding ethnic minority membership and fear of crime. It is noteworthy that the main effect was non-significant, that is, the average effect across all countries. This demonstrates the importance of examining cross-level interactions, which clearly showed that the association between ethnic minority membership and fear of crime differed depending on the societal context. Surprisingly, it is not ethnic minority members but ethnic *majority* members who report greater fear of crime in unequal societies. This raises the question of what may underlie a fear that is specific to ethnic majority members. One possibility is that the majority is afraid of crime committed by ethnic *minorities*. This is somewhat consistent with an earlier and smaller cross-national ESS study on perceived immigrants’ impact on crime in which the results suggested that people’s fear of crime may be a disguised form of prejudice against foreigners (Ceobanu, 2010). It is noteworthy that fear of crime committed by immigrants was not affected by the national wealth of a country (as assessed through Gross Domestic Product measures). In a similar vein, we did not find the HDI to be related to fears of crime, which points to the importance of *relative* deprivation (as assessed through income inequality) as opposed to absolute deprivation. It supports our reasoning that income inequalities foster a climate of fear and general mistrust in others because there is a shared awareness that there are very limited legitimate options for the disadvantaged to obtain resources and social status. Hence, there is a constant feeling of threat that is not necessarily matched by a real threat (for a similar argument, see Bratanova et al., 2016). For instance, Ceobanu (2010) found that the ratio of foreign inmates to the non-European foreign population was unrelated to public views about immigrants’ impact on crime (Ceobanu 2010), yet perceptions that immigrants worsen crime problems were more evident in societies harbouring larger stocks of non-European immigrants. This is fully compatible with the argument that unequal societies are more prejudiced (Vauclair et al., 2015; Wilkinson and Pickett, 2009). Generally, there is a greater orientation towards hierarchy and social dominance in unequal societies and therefore any low-status group members are more likely to be evaluated negatively, resulting in social exclusion and discrimination (Marmot, 2004). Therefore, it could be the case that one way of managing and negotiating threats and dangers in these societies is to resort to stereotyping and prejudice. By labelling criminals with certain social identifiers (for example foreigners), a sense of control is restored (Smith, 1984). In this sense, the fear of crime measures may be less nurtured by actual fear of crime and more by a fear of foreigners. This may be fuelled by perceptions of overlap between the notions of immigrant and outlaw, the limited professional opportunities for foreigners that marginalize them in society, and that some immigrants come illegally or overstay their visa (see Ceobanu, 2010). Future research may examine the phenomenon of racial crime prejudice in unequal societies more deeply.

We also aimed to test a mediation model in which fear of crime is the mediator that explains the inequality–subjective well-being link. Although all associations in our model were significant in the expected direction, fear of crime could not explain this link in the general population. Our follow-up analyses showed that this might be owing to the fact that income inequality is not related to greater fear of crime for ethnic *minority* members. The mediation model held only in the subpopulation of ethnic *majority* members. This is still the large majority of the representative samples in each country, and therefore we may conclude that fear of crime explains why income inequality leads to lower levels of subjective well-being for most of the people in a country. As pointed out above, these findings merit further investigation in order to fully understand the nature of fear in this subpopulation.

There are some limitations to consider when interpreting our results. First, it is important to recognize that our study is cross-sectional and therefore does not allow us to draw definite conclusions about cause and effect. Yet it seems reasonable to assume that the macro-variable national income inequality is not primarily caused by fear of crime or subjective well-being. Hence, the cause and effect question is predominantly about the association between fear of crime and subjective well-being. It might be that individuals who are not well are also more fearful of crime. However, the literature usually conceptualizes subjective well-being as a consequence of fear of crime (for example, Morrall et al., 2010). In reality, a complex bidirectional relationship may apply. The analyses of cross-national longitudinal data could shed some light on the cause and effect issue. However, this type of data is not available for the ESS.

Secondly, we should be cautious about the generalizability of these findings to other regions of the world. The data are representative of countries within the European region, so they do not necessarily generalize to other regions or continents. Moreover, the sample size at the country level is relatively limited, which means that the tests of country-level effects may have been underpowered. Nevertheless, income inequality emerged as a robust predictor of fear of crime even when controlling for other relevant country-level variables. Studies examining fear of crime or related constructs with cross-national data from the European region usually operate with low sample sizes (between 21 and 27 countries, see Ceobanu, 2010; Hummelsheim et al., 2011; Semyonov et al., 2012; Vieno et al., 2013; Visser et al., 2013). By using a multilevel model, we made the assumption that our clusters can be regarded as a random sample from a wider population, allowing us theoretically and statistically to infer our results beyond the countries that were used in the analysis. Considering that there are other relatively wealthy regions and countries in the world with an even greater discrepancy in the distribution of income (for example, in the US – OECD, 2008), it is plausible that there are similar or possibly even stronger relationships when the model is tested on a more diverse national or international dataset. For instance, income inequality differs considerably across the US states and it has already been suggested that this should foster individuals’ fear of crime if they live in highly unequal states (Wilkinson and Pickett, 2009).

Third, one limitation of using macro-level indicators as predictors of fear of crime is that there is some ambiguity left as to how exactly the context affects individuals’ fears of crime. We drew upon the income inequality literature and theorized that a strong division between the rich and the poor renders social mobility in a hierarchical society more unlikely. This fosters a climate of fear because people believe that there are few legitimate chances for the deprived to obtain resources and social status, leaving crime and violence as the most obvious route – even if this is not necessarily the case. This can also be seen as a sign of mistrust in others, which is consistent with our results reported above. Hence, inequality is a structural aspect of societies that gets under the skin by having a relative income, social status or class position in the wider societal fabric (Wilkinson and Pickett, 2009).

Further research is needed in order to ascertain the specific pathways of how income inequality fosters fear of crime. Such a model should treat fear of crime as the criterion variable, national income inequality as the independent variable, and individual-level characteristics, such as perceptions about inequality, as mediator variables. Such an analysis would make a significant advancement to the fear of crime literature and its link to the macro-level variable income inequality. We also acknowledge that the explained variance at the country level for income inequality as the only predictor does not appear to explain much between-country variance (about 17 percent), leaving the question of whether this contextual effect is relevant enough or whether the meso-level (for example, neighbourhood characteristics) is more useful to study since it is also more proximate to individuals’ living environment. We think that the meso-level is indeed highly relevant for this topic of research; however, we would like to emphasize that, even if macro-level contextual effects emerge as relatively small, they are important from a theory and policy point of view. The reason is that these effects should operate over a wide range of research areas and dependent variables (Liska, 1990). Research on income inequality has already yielded numerous important findings in how it is related to a host of social and health problems (see Wilkinson and Pickett, 2009). This cumulative evidence – including that provided by the current study – is helpful to make a case for reducing income inequalities and therefore alleviating at once many of the social and health problems societies are facing today.

## Notes

1. Another common meso-level characteristic that has been linked to fear of crime is the spread of visible signs of disorder (for example, unsupervised teenage groups, littering and graffiti; see, for example, Wilson and Kelling’s 1982 ‘broken windows’ theory). Yet we are unaware of any empirical study that has examined this environmental characteristic at the country level, probably owing to the fact that there is a lack of data representing signs of disorder at the aggregated country level.

2. URL (accessed 26 April 2016): http://appsso.eurostat.ec.europa.eu/nui/show.do?dataset=ilc_di12&lang=en.

3. URL (accessed 26 April 2016): http://www.wider.unu.edu/research/Database/.

4. URL (accessed 26 April 2016): http://appsso.eurostat.ec.europa.eu/nui/show.do.

5. This variable was assessed through two items (0 = ‘you can’t be too careful’ to 10 = ‘most people can be trusted’; 0 = ‘most people try to take advantage of me’ to 10 = ‘most people try to be fair’). The inter-item correlation was satisfactory, ranging from .42 (France) to .73 (Greece). We computed an overall index of generalized trust by averaging the scores and aggregating them at the country level.

6. We are not aware of any cross-national study that used the degree of urbanization as a predictor variable yet, drawing upon the meso-level studies (see Pain, 2000), it seems conceivable that a higher level of urbanization could be related to greater fear of crime.

7. All indirect effect confidence intervals are 90% to correspond to the one-tailed, α = .05 hypothesis test (see Preacher, et al. 2011).
